# Surface Adsorption Mechanism between Lead(II,IV) and Nanomaghemite Studied on Polluted Water Samples Collected from the Peruvian Rivers Mantaro and Cumbaza

**DOI:** 10.3390/nano13101684

**Published:** 2023-05-20

**Authors:** Juan A. Ramos-Guivar, Noemi-Raquel Checca-Huaman, F. Jochen Litterst, Edson C. Passamani

**Affiliations:** 1 Grupo de Investigación de Nanotecnología Aplicada Para la Biorremediación Ambiental, Energía, Biomedicina y Agricultura (NANOTECH), Facultad de Ciencias Físicas, Universidad Nacional Mayor de San Marcos, Av. Venezuela Cdra 34 S/N, Ciudad Universitaria, Lima 01, Peru; 2 Centro Brasileiro de Pesquisas Físicas, Rio de Janeiro 22290-180, RJ, Brazil; noemiraquelchecca@gmail.com; 3 Institut fur Physik der Kondensierten Materie, Technische Universität Braunschweig, 38106 Braunschweig, Germany; j.litterst@tu-braunschweig.de; 4 Departamento de Física, Universidade Federal do Espírito Santo -UFES, Vitória 29075-910, ES, Brazil; edson.caetano@ufes.br

**Keywords:** lead removal, maghemite nanoadsorbent, surface adsorption mechanism, natural polluted waters

## Abstract

Real water remediation is an important issue that requires the development of novel adsorbents with remarkable adsorption properties, permitting reusability. In this work, the surface and adsorption properties of bare magnetic iron oxide nanoparticles were systematically studied, before and after the application of a maghemite nanoadsorbent in two real Peruvian effluents severely contaminated with Pb(II), Pb(IV), Fe(III), and others. We were able to describe the Fe and Pb adsorption mechanisms that occurred at the particle surface. ^57^Fe Mössbauer and X-ray photoelectron spectroscopy results together with kinetic adsorption analyses gave evidence for two involved surface mechanisms: (i) surface deprotonation of maghemite nanoparticles (isoelectric point of pH = 2.3), forming Lewis sites bonding Pb complexes; and (ii) the formation of a thin inhomogeneous secondary layer of iron oxyhydroxide and adsorbed Pb compounds, as favored by surface physicochemical conditions. The magnetic nanoadsorbent enhanced the removal efficiency to values of ca. 96% and provided adsorptive properties with reusability due to the conserved morphological, structural, and magnetic properties. This makes it favorable for large-scale industrial applications.

## 1. Introduction

The surface and adsorption properties of magnetic nanoparticles (NPs) deserve scientific investigations concerning the treatment of real effluents (rivers). Particularly, water bodies become continually strained by toxic metals, such as lead (Pb), cadmium (Cd), and chromium (Cr), a pollution problem that may have natural and/or anthropogenic origins, the latter mainly due to agriculture and mining activities [1].

In South America [2], legal and illegal mining activities produce a gradual and continuous reduction in biodiversity and also accumulate tailings. Usually, the contaminations are obtained through mineral flotation processes, in which the valuable mineral is severely extracted from the rest, causing, therefore, an increase in the degree of contamination in the water receptors that are often lakes, lagoons, rivers, and the sea. Therefore, the diffusion of toxic metals into water bodies provokes a progressive and constant accumulation, affecting both living organisms and the environment.

It is unnecessary to emphasize that water is essential for all living beings, but the available fresh drinking water is only 2.5% of the global fraction when one compares it with the sea and ocean waters that have an amount ca. 97%. Therefore, regarding water contamination, illegal mining in several developing countries (e.g., Peru, in particular) is partly responsible for the short- and long-term exposure and damage to the water bodies with a high degree of toxic metals that certainly alters their ecosystems [3]. One of the most common contaminants from Peruvian mines is Pb (mainly in the form of ions and compounds), a toxic metal that has the greatest human health risk when its ingestion and exposure occurs above its WHO permissible level of 10 µg L^−1^ [4]. It may cause learning difficulties in children, consequently affecting their intellectual development [5]. In adults, cardiovascular diseases [6] and genotoxic effects in several human organs [7] have been reported. Thus, considering (i) the unfortunate situation of several towns in developing countries close to mining activities and (ii) that the people living nearby are practically forced to ingest these polluted waters, it is urgently necessary to develop processes to remove and/or reduce Pb(II) contaminants quickly and effectively to make the waters of the ecosystems suitable for human consumption, animal nutrition, and also irrigation.

In response to this issue, various ordinary methods have been applied for purifying Pb-contaminated water [8,9,10]. The adsorption processes represent one of the latest trends in water purification, without adverse effects on health, being highly effective for decontamination [11,12]. We highlight banana peel, a natural system that has been applied for removing Pb from a synthetic effluent [13]. Another natural adsorbent that has been tested is based on the exoskeletons of shrimps [14]. Synthetic adsorbents, e.g., inorganic magnetic iron oxide nanoadsorbents, have also been applied, with a Pb removal efficiency of 99.9%, short equilibrium time of only 5 min, and fast magnetic separation response [15].

Here, it should be mentioned that an improvement in the adsorbent properties has been observed using a nanohybrid composed of magnetic nanoparticles (NPs) and zeolites 5A, an effect that was explained considering an increase in the specific surface area, active sites, and magnetic response of the resultant nanohybrid’s mesoporous material. These properties open perspectives towards 100% Pb(II) removal via an adsorption process dominated by cation exchange among calcium ions, the protonated maghemite (γ-Fe_2_O_3_) surface, and Pb cations. Therefore, new nanohybrids using other functional groups (organic and inorganic) have also been developed and tested [12,15,16]. Hence, depending on the adsorbent nature, many synergetic adsorption mechanisms can occur, including electrostatic attraction, chelating, cation exchange, host–guest interaction, and others [17].

Regarding the excellent results of Pb removal reported in the literature [8,9,10,11,12,13,14,15,16], most of the reported works tested synthetic effluents, where the ambient of the effluent was well controlled and the effects of other contaminants were mostly discarded. The adsorption experiments are often performed only for Pb(II) species. However, in real waters, other Pb species and other constituents are found, which may affect the Pb(II) adsorption [14,15,16,17]. Hence, the adsorbents must be re-evaluated under this condition, assuming their applications for the magnetic remediation process. For instance, for pure magnetic NPs, the complexation between iron oxide NPs and Pb chemical groups is still less studied for understanding the complete surface adsorption mechanism.

The above-mentioned aspects were issues in the present work. In particular, we synthesized γ-Fe_2_O_3_ NPs using the conventional co-precipitation method, and systematic structural and adsorption characterizations were performed using several analytical techniques, including X-ray diffraction, N_2_ physisorption, infrared and Raman spectroscopy, and transmission electron microscopy. The Pb removal properties were evaluated for water samples collected from two Peruvian rivers, and their kinetic adsorption mechanisms were investigated. To understand the surface adsorption mechanism, transmission electron microscopy (TEM), energy-dispersive spectroscopy (EDS), X-ray photoelectron spectroscopy (XPS), ^57^Fe Mössbauer spectrometry, and vibrating sample magnetometry (VSM) were systematically performed on the samples after Pb removal. Thus, this study brings information about the surface properties of pure γ-Fe_2_O_3_ NPs and their interactions with Pb ions in contaminated effluents obtained from real rivers, an issue that has been rarely discussed in the literature.

## 2. Materials and Methods

### 2.1. Adsorbent Synthesis

For the adsorbent synthesis, the chemical co-precipitation method was used. For this, 250 mL of distilled water was added to a flat-bottomed flask. Then, it was placed on a magnetic stirrer, with a stirring speed of 550 rpm, until it reached a stable temperature of 80 °C. After that, 12.0 g of FeCl_3_ and 10.4 g of FeSO_4_•7H_2_O were added to the heated water, upon which a reddish dispersion was obtained and kept at 80 °C. Immediately after, 50 mL of NH_4_OH was added to the solution under vigorous stirring for 30 min. Then, the reaction product was left to cool down to room temperature (RT), and a neodymium-based magnet was used to assist the washing procedure until a solution with pH = 7 was obtained. Subsequently, the sample was dried in an oven at 80 °C for 6 h and the final powder was labeled as RG1.

### 2.2. Characterization

Cu-K_α_ X-ray diffractogram was recorded for the RG1 sample with Empyrean equipment (Malvern Panalytical, Malvern, UK) under the same conditions reported in the literature [15,16]. The X-ray diffractogram (XRD) was refined using the Rietveld method, using the FullProf Suite program, in which the Al_2_O_3_ phase was considered as standard. The structural identification was carried out using the CIF # 9005814 given by the Match! Program v3 [13]. The Thompson–Cox–Hastings (TCH) function was used to perform diffraction profile modeling. N_2_ adsorption/desorption isotherms recorded at 77 K were measured using a Micrometrics Tristar 3000 Sorptometer. Before measurements, a degasification step was performed at 300 °C for five hours. Textural parameters, including specific surface area and pore size distribution, were, respectively, estimated using the BET and BJH models, where the pore volume was considered at P/P_0_ = 0.98. The RT infrared (IR) spectrum data were collected by using an IRPrestige-21 Shimadzu spectrophotometer. For this analysis, the IR frequency range varied between 400 and 4000 cm^−1^, considering an optical resolution of 2 cm^−1^. The Raman characterization was carried out using a Renishaw inVia Raman microscope. A Helium–Neon laser with wavelength of 633 nm and initial laser power of 7.17 mW was employed as excitation source. The total exposure time of the samples was two accumulations of 10 s each, with an objective of 50× magnification for measurements. Several fractions (%) of the initial laser power were used to systematically analyze the Raman spectra using 0.05%, 0.1%, 0.5%, 1%, 5%, 10%, and 50%, respectively. This process was labeled as “before burning protocol”. The “after burning protocol” was performed by first exposing the sample to 7.17 mW and repeating the steps for “before burning experiments”. The titration curve of the dispersed sample was determined using a Brookhaven Nanobrook 90 Plus PALS. The initial suspension pH value was 7. For determination of average particle size, histogram distributions, and NP morphologies, the electron imaging (EM) microscopy technique was applied under three operative modes, i.e., in transmission (TEM), in scanning (STEM), and in transmission at high-resolution (HRTEM), which are available in the JEOL 2100F (Tokyo, Japan) instrument working at an accelerating voltage of 200 kV and using a CMOS camera for acquisition of TEM and HRTEM images. STEM images were acquired using a high-angular annular dark-field (HAADF) field detector. The microscope was also equipped with accessories for energy-dispersive X-ray spectroscopy (EDS). The elemental compositions of the samples were investigated via EDS to evaluate the atomic composition in STEM mode. The ^57^Fe Mössbauer spectra recorded at 15 K, 17 K, and RT (300 K) were collected in transmission mode using a conventional spectrometer operating with sinusoidal velocity sweep and using a 40 mCi source of ^57^Co in Rh matrix. For the low-temperature measurements, the source was kept at RT, and the absorber temperature was decreased to 15 K, employing a Janis closed-cycle setup. The powder absorber was put into nylon sample holders, and its effective thickness was chosen to be equivalent to ca. 0.1 mg ^57^Fe per cm^2^. Finally, magnetic properties were studied using zero-field-cooling (ZFC) and field-cooling (FC) protocols. Thus, *M(H)* loops were collected at RT and 5 K using a vibrating sample magnetometer (VSM) working in a dynacool setup with a maximum field ($B=\mu_{o}H$) of 7 T. The FC experiment was conducted with a cooling field of 1 T and a sweep field of ±7 T (the FC experiments were carried out to check for possible exchange bias effect).

### 2.3. X-ray Photoelectron Spectroscopy before and after Heavy Metal Adsorption Experiments in Real Waters

Chemical surface analyses on the studied samples were performed via X-ray photoelectron spectroscopy (XPS) using the SPECS PHOIBOS 100/150 equipment with a hemispheric analyzer spectrometer, operating at 1486.6 eV of Al Kα. The XPS spectra were collected with a high-resolution polychromatic X-ray source within an energy step of 0.02 eV. Casa-XPS software (SPECS company) was used to fit the peak positions of the Fe 2p, Pb 4f, Si 2p, O 1s, and C 1s levels to determine the chemical binding energies (BEs) of formed species and to calculate the relative atomic quantities on the sample surfaces. The spectra were calibrated using adventitious carbon (B.E. reference) at C 1s = 284.6 eV, previously using an electron flood gun at 12 µA and 1 eV.

### 2.4. Adsorption Kinetic Protocol in Real Waters

The collection of real waters from the Mantaro River (Junin department, coordinates 12°16′0″ S 73°57′0″ W, 12:20 p.m. 1 August 2021) and the Cumbaza River (San Martin department, coordinates 6°36′5″ S 76°19′46″ W, 10:49 a.m. 5 October 2021) was carried out following a standard sampling procedure: (i) water was stored in a 20 L container that was previously rinsed three times with ultrapure water; (ii) it was collected from the surface of rivers to avoid the presence of sediments, and (iii), subsequently, the aliquots were stored using nitric acid (HNO_3_). The Pb concentrations detected by inductively coupled plasma optical emission spectroscopy (ICP) were 0.19 and 0. 28 mg L^−1^, respectively, for Mantaro and Cumbaza rivers, values that are 19- and 28-times higher than the permissible level reported by the WHO (10 µg L^−1^) [4]. The initial pH value in both rivers varied from 6.5 to 7 on the days that the samples were collected. Different Pb species will coexist in a real water environment. Hence, to ensure the presence of Pb(II) species, it was necessary to modify the concentration using lead nitrate (Pb(NO_3_)_2_). The modified concentrations were 1.7 mg L^−1^ (Mantaro, modified pH equal to 7.8) and 18 mg L^−1^ (Cumbaza, pH equal to 6.8). Additionally, other toxic metals, such as Mn, Fe, and Cd, were quantified since they are known to interfere with Pb(II) adsorption [12]. For the Mantaro river, the concentrations of Mn, Fe, and Cd were, respectively, equal to 0.01 mg L^−1^, 0.9 mg L^−1^, and Cd 0.009 mg L^−1^. For the Cumbaza river, the concentrations were found to be 0.01 mg L^−1^ Mn, 0.21 mg L^−1^ Fe, and 0.009 mg L^−1^ Cd. Mn concentration in both rivers was below the value reported by the WHO as a permissible level [18], while the Fe concentration exceeded the permissible level in the Mantaro river [18]. In addition, the Cd concentration was three-times higher than the value of the permissible level for drinking water (3 µg L^−1^) in both rivers [18].

For batch kinetic experiments, 25 mg of adsorbent was added to 50 mL of initial Pb solution at 25 °C for intervals of 5 min to 24 h. Once the equilibrium time was determined, the adsorbent dose at RT was investigated by changing the adsorbent amount (5, 10, 15, 25, 30, and 50 mg) in contact with Pb initial solution from the Cumbaza river only. The initial and equilibrium pH values were monitored in all kinetic experiments. The quantification of Pb concentrations was performed using atomic absorption spectroscopy. A Varian atomic absorption spectrometer was used for measurements after calibration with stock solutions. To estimate the total lead amount, we employed the difference between the initial (*C*_0_) and final (*C_t_*) concentrations (mg L^−1^) in the solutions, which is mathematically expressed as [15]:

(1) $$q_{t}=\frac{\left(C_{0}-C_{t}\right)V}{m}\;\;\;\;(\mathrm{mg}\;g^{-1})$$

where $q_{t}$ is the adsorbed amount per gram at certain time *t*; *m* is the adsorbent mass (g); and *V* (L) is the batch volume. The homogenization time between the magnetic adsorbent RG1 and the Pb(II) solution occurred after 15 min of prior stirring. After that, the kinetic adsorption time was measured at different times. After each adsorption kinetic experiment, the solid part of the solution was separated. For this purpose, a cylindrical neodymium-based magnet was employed and placed near the solution for 15 min to magnetically separate the solid part from the resulting solution. The recovered mass used further for adsorption experiments (Cumbaza river only) was labeled as RG2. The samples recovered after the adsorption kinetic experiments were labeled as RG3 (Cumbaza river) and RG4 (Mantaro river), respectively. In this manuscript, this series of samples will hereafter be named as RGx (x = 1–4).

## 3. Results and Discussion

### 3.1. X-ray and Rietveld Analysis

**Figure 1:**
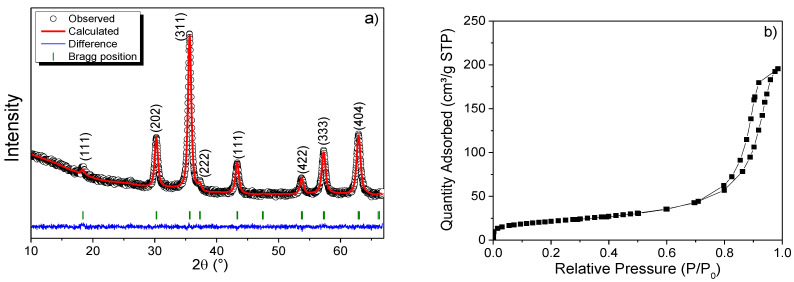

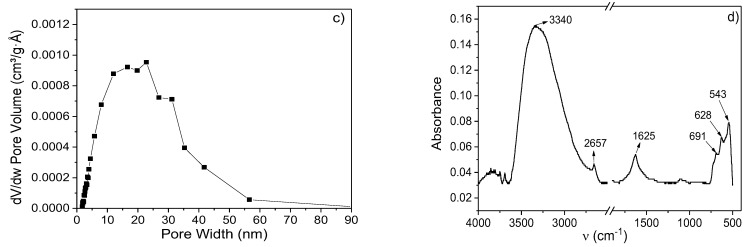
(**a**) Refined diffractogram, (**b**) N2 adsorption–desorption obtained at 77 K, (**c**) pore size distribution, and (**d**) IR absorbance spectrum for the RG1 sample.

The Rietveld refinement of the X-ray diffractogram of the RG1 sample is shown in Figure 1a. Table 1 summarizes the refined parameters. Structurally speaking, the theoretical model agrees with the cubic inverse spinel structure, space group Fd$\bar{3}$m. However, it should be mentioned that it is extremely hard to differentiate between Fe_3_O_4_ and γ-Fe_2_O_3_ structures from X-ray experiments because both crystal phases are quite similar on the nanoscale [12]. On the other hand, regarding the mean crystallite size, the RG1 nanoadsorbent had a value of 15.2(3) nm, a size that corroborated with the nanoscale character of the RG1 sample (more information about particle size will be given with TEM data below).

### 3.2. Textural Properties

Figure 1b shows the hysteresis adsorption isotherm recorded at 77 K for the RG1 sample. The estimated textural properties were BET area of 75.9 m^2^/g, pore volume of 0.298 cm^3^/g, and pore diameter equal to 15 nm. As observed from Figure 1c, the pore distribution (estimated from BJH method) revealed a broad pore distribution. The shape of the isotherm curve was characteristic of an IV-type isotherm related to mesoporous materials [19].

### 3.3. IR and µ-Raman Analysis

In the low-IR region, three characteristic peaks were found, located at 543, 628, and 691 cm^−1^ (see Figure 1d). According to Vidal-Vidal et al. [20], the optical IR absorption bands related to Fe^3+^–oxygen stretching vibration are positioned at 750 to 550 cm^−1^ for tetrahedral Fe sites and from 550 to 300 cm^−1^ for octahedral Fe sites. They are assigned to the cubic spinel structure of the γ-Fe_2_O_3_ phase [20]. No Fe^2+^–oxygen stretching vibrations were observed. Between 1625 cm^−1^ and 3340 cm^−1^, a broad IR band was seen that may be related to the bending vibration of water molecules δ (OH) and stretching vibration of OH groups [21]. However, the differentiation about Fe^2+^ and Fe^3+^-oxygen coordination could not be taken as conclusive since IR experiments are sensitive to Fe site deficiency and transitional symmetry lost in the γ-Fe_2_O_3_ NPs. It means that IR modes of the γ-Fe_2_O_3_ phase can overlap to activated IR modes of the Fe_3_O_4_ [22] and they can be masked in the case of core–shell Fe_3_O_4_@γ-Fe_2_O_3_ (it is difficult to differentiate both inverse spinel lattices).

On the other hand, in a typical Raman measurement, careful analysis must be conducted when analyzing iron oxide NP samples. An increase above the threshold laser power [23] might lead to a misunderstanding of an analyzed iron oxide phase due to structural alterations that may be enhanced in NP, i.e., a structural phase transformation: γ-Fe_2_O_3_→α-Fe_2_O_3_ (or simply γ→α). Previous studies have shown that the threshold value depends strongly on the size of the NPs and also the functionalized agent. The latter may give thermal stability to the iron oxide NP sample, i.e., the γ→α transition will occur at higher temperatures [23,24,25,26]. Hence, two protocols given in the experimental sections were performed to correctly analyze the RG1 sample.

#### 3.3.1. Characterization of Sample RG1 (“before Burning Protocol”)

As previously mentioned, the γ-Fe_2_O_3_ and Fe_3_O_4_ phases were hardly differentiated by several experimental methods when these materials were in the nanoscale regime. Regarding IR spectroscopy, Fe_3_O_4_ had three well-defined Raman bands, with a characteristic intense optical mode located at 670–680 cm^−1^ (A_1g_) [22,23], i.e., this mode is more intense (than the other two active optical modes) and can be used to identify the Fe_3_O_4_ phase, as will be discussed below.

**Figure 2:**
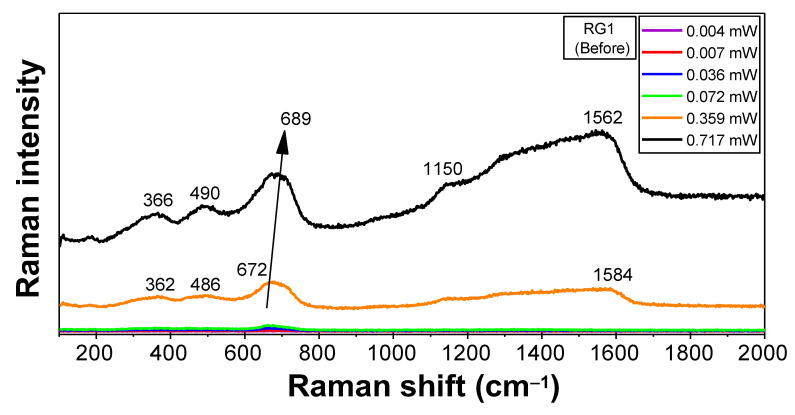
Raman spectra of the RG1 sample recorded at several laser powers. The arrow indicates the main optical Raman mode usually found in γ-Fe_2_O_3_ NPs.

On the other hand, the γ-Fe_2_O_3_ phase also exhibited three featured Raman modes, but differing from those of the Fe_3_O_4_ phase, because the main optical mode had a broadened signal slightly shifted to 720 cm^−1^ [23]. Specifically, uncoated γ-Fe_2_O_3_ NPs had more Raman optical modes, and, in particular, the third peak can be deconvoluted into 678 cm^−1^ (E_g_) and 723 cm^−1^ (A_1g_) [27]. However, for pure Fe_3_O_4_ NPs, the additional Raman mode at ~720 cm^−1^ is absent. Hence, the apparent absence of this optical active mode may help us to differentiate samples containing the pure γ-Fe_2_O_3_ or Fe_3_O_4_ phase. It is also important to highlight that on the nanoscopic scale, Raman spectra depend on the particle size and thermal, chemical, and aging effects, sometimes leading to a broadening effect of Raman bands. In particular, the Fe_3_O_4_ NPs, exposed to normal air conditions, tended to oxidize superficially, consequently forming a core–shell arrangement, where the shell was mainly composed of the γ-Fe_2_O_3_ phase. This process makes it tough to identify the Raman bands of Fe_3_O_4_ NPs. One way to solve the presence of the γ-Fe_2_O_3_ shell is exposing the sample to different laser powers, because the γ-Fe_2_O_3_ phase must exhibit extra Raman modes when the structural phase transformation to α-Fe_2_O_3_ occurs. This phenomenon may be related to the local temperature change produced by the laser power that may reach values above the Curie temperature (580 °C) of the γ-Fe_2_O_3_ phase [23,24,25]. Thus, the Raman spectra (0.004 mW to 0.717 mW) for the RG1 sample are recorded and plotted in Figure 2.

The Raman spectrum of the RG1 sample has a fingerprint associated with the Fe_3_O_4_ signal at 672 cm^−1^ up to a laser irradiation power of 0.359 mW, whilst the fingerprint signal for the γ-Fe_2_O_3_ phase should occur at 720 cm^−1^ [23,27]. Upon an increase in laser power, we found a slight shift in the Raman peak from 672 cm^−1^ to 689 cm^−1^, indicating the formation of the γ-Fe_2_O_3_ phase at the Fe_3_O_4_ NPs surfaces. These results support an Fe_3_O_4_-γ-Fe_2_O_3_ core–shell-type scenario, a supposition that is supported by the “after burning protocol”, as will be shown below. A γ→α Fe_2_O_3_ phase transformation was not observed in the sample under the tested laser powers; therefore, the γ-Fe_2_O_3_ shell thickness of the sample is much smaller than the fraction of the Fe_3_O_4_. However, this state may gradually change over time due to exposure to normal atmospheric conditions or under even higher laser power values.

#### 3.3.2. Characterization of Sample RG1 (“after Burning Protocol”)

The RG1 sample was intentionally burned at 100% at a power of 7.17 mW for 20 s and the hematite phase (α-Fe_2_O_3_) was easily observed, as displayed in Figure S1. Therefore, the γ-Fe_2_O_3_ fraction initially presented at the surface of the Fe_3_O_4_ NPs gradually increases in amount with time and laser exposition. As a consequence, the α-Fe_2_O_3_ phase is noticeably present after annealing the RG1 sample, starting at a power of 0.359 mW and better identified by gradually increasing the laser power during the IR experiments. Three mechanisms are proposed to describe the gradual transformations mentioned above: (i) the part of the shell surface composed of γ-Fe_2_O_3_ first transforms to α-Fe_2_O_3_ during laser exposure, releasing heat and oxygen, (ii) the released oxygen leads to the oxidation of nanomagnetite (Fe_3_O_4_+O_2_ → γ-Fe_2_O_3_), and (iii) this nano γ-Fe_2_O_3_ follows the process of structural phase transformation to α-Fe_2_O_3_ according to (i).

Considering that the NPs synthetized through co-precipitation were all acceptable, the Pb removal experiments can now be discussed assuming that the main constituent of the core of the NPs is basically formed by γ-Fe_2_O_3_ (the as-prepared NPs were applied months later and the Fe_3_O_4_ → γ-Fe_2_O_3_ transformation naturally occurred, as will be shown by other results).

### 3.4. Pb Removal Efficiency in Real Waters

**Figure 3:**
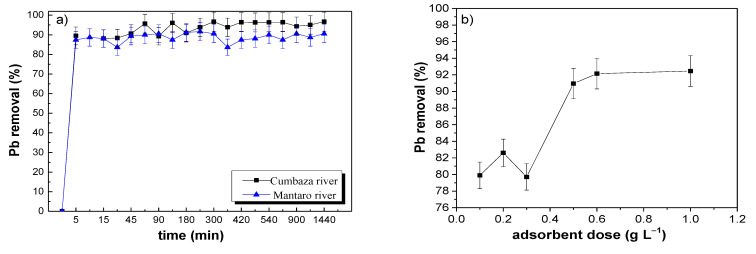

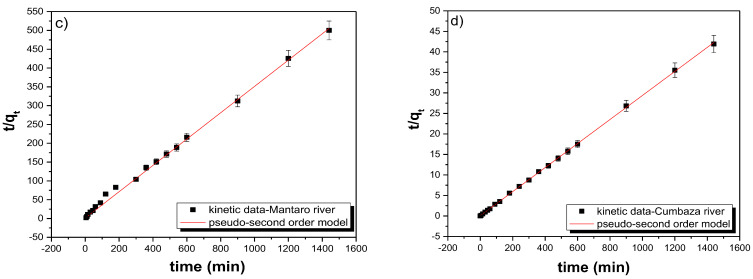
(**a**) Total Pb removal efficiency vs. time, (**b**) total Pb removal efficiency vs. RG1 adsorbent dose, (**c**,**d**) linear fits for the pseudo-second-order model of data recorded from the Mantaro and Cumbaza rivers.

The dependence of the toxic Pb metal uptake efficiency versus time is shown in Figure 3a. Both experiments performed with effluents from the Mantaro and Cumbaza rivers indicated a Pb removal better than 90%. In particular, for the Mantaro River, a removal percentage of 90% was obtained in an equilibrium time of 10 min, whereas the equilibrium time occurred for 60 min with a maximum removal percentage of 96% for the Cumbaza river. When comparing to other adsorbent materials based on nanoscopic Fe-oxides [12], the RG1 sample is significantly competitive. The proposed adsorbent system also presents results in two real water bodies compared to the systems reported in the literature, which are usually carried out in synthetic waters (i.e., simulated waters containing no interferents) [12,13]. The Fe concentration after adsorption studies (using the RG1 sample, in both rivers) is below the limit of detection of the equipment (<10 µg L^−1^). In the case of Mantaro river, the equilibrium time was 5 min for Fe, whilst for the Cumbaza river, a value of 8 h was reached. This means that Fe ions can also be removed using the RG1 adsorbent. More importantly, Fe ions are not leached during the adsorption process. In the case of Cd concentrations, the values remained in the same range after the Pb removal, which indicated that the RG1 sample was not chemically interacting with this divalent toxic metal, as will also be shown by the XPS results.

### 3.5. Kinetic Adsorption Models

A detailed analysis of adsorption kinetics was performed using four kinetic adsorption models [15]: (i) pseudo-first-order (PFO) kinetic model, (ii) pseudo-second-order (PSO) kinetic model, (iii) intraparticle diffusion kinetic model, and (iv) the Elovich kinetic model. The best fits of experimental data were achieved for the PSO kinetic model, as shown in Figure 3b,c, for both rivers. The corresponding fit parameters are summarized in Table 2.

The fit parameters in the PSO model are related to the chemisorption process, i.e., the process is dominated by chemical binding. It is worth mentioning that the other kinetic models (data not shown) tested in our experimental data suggest that they seem to not work in real water bodies, as indicated by unreasonable R^2^ values, usually below 0.5.

### 3.6. Effect of Adsorbent Dose on Pb Removal

From the adsorption kinetics process in the Cumbaza River, it was found that the equilibrium time for the RG1 nanoadsorbent was 60 min. Figure 3d shows how Pb(II) removal varied at selected doses. As a result, the RG1 nanoadsorbent reached a maximum value of 93% for 1 g L^−1^. After that point, the curve reached a plateau that marked the maximum adsorbent mass significant for Pb removal.

### 3.7. pH Monitoring during the Adsorption Kinetic and Adsorbent Dose Processes

The pH value in a Pb solution prepared in distilled water has a value of 5.5 [15]. However, as shown in Figure S2, it was observed that when varying the concentration of Pb in the real waters, the initial pH was 7. However, after each time exposure to the RG1 sample for the kinetic experiments in both rivers, the pH values (obtained with a pH meter) fluctuated between 6.5 and 7.5. The same results were obtained when changing the adsorbent dose in the case of the Cumbaza river. These results suggest that it is not necessary to significantly vary the pH of real effluents to achieve the Pb adsorption process using the RG1 sample. Thus, this result is a gain against the usually used Pb removal process with commercial acids in order to clean up 100% of Pb(II) at pH = 5.5 [12]. Thus, the results presented up to this section indicate that our nanoadsorbent is highly efficient in removing total Pb in real water, even if one considers it at scalable industrial levels.

To better understand the chemical adsorption of Pb on the NP surfaces, it should be mentioned that the surface chemical configuration available in γ-Fe_2_O_3_ will depend on the point of zero charge (p.z.c.) that is located at pH = 2.3 for the RG1 sample, as shown in Figure S3. Above this value, the outer sphere complex between Pb(II) and surface chemical groups of γ-Fe_2_O_3_ can be favored. Therefore, it is assumed that the following deprotonation reaction will occur [28]:

(2) $$\equiv \mathrm{Fe}-\mathrm{OH}⇄\equiv \mathrm{Fe}-O^{-}+H^{+}\;\;\;\;(>\;pH_{p.z.c.})$$

The last $\mathrm{Fe}-O^{-}$ group coordinates with Pb(II) forming $\mathrm{Fe}-O-{\mathrm{Pb}}^{+}+H^{+}$ complexes. This can explain the remarkable adsorption for Pb(II) at a pH ranging from 6.5 to 7.5 (negative zeta potential of ~−17 mV).

In brief, all the above surface sorption mechanisms can be fully explained by the interaction between the RG1 sample and Pb(II) cations. However, it should be kept in mind that the total removal also implies the presence of other Pb cation species commonly found in real waters.

### 3.8. Main Experimental Results Obtained after Adsorption Analysis

#### 3.8.1. TEM and EDS Analysis

**Figure 4:**
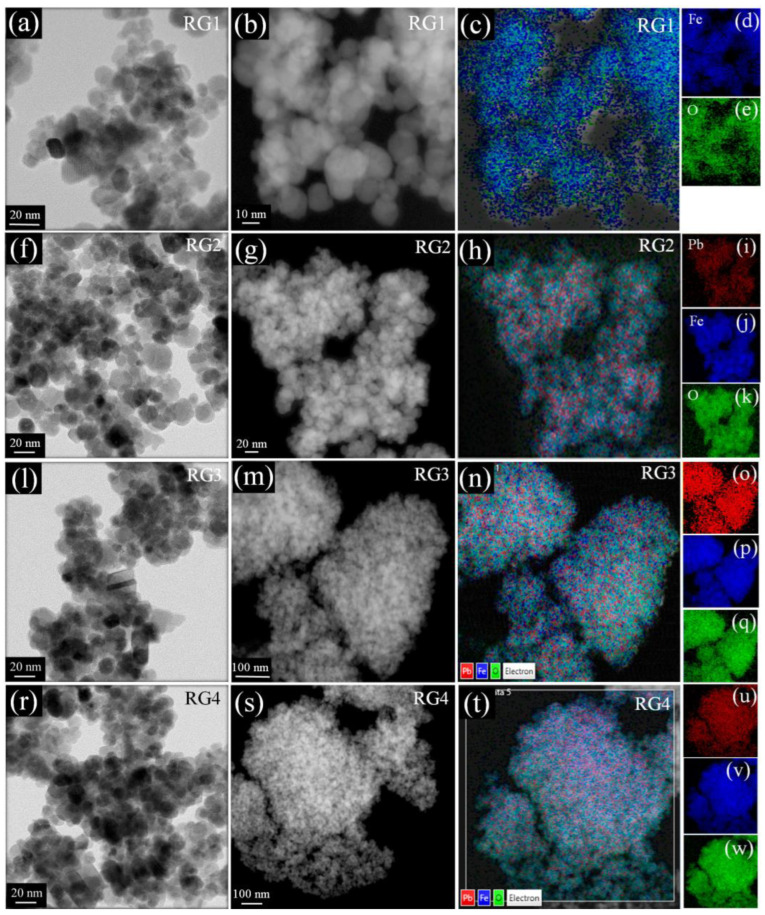
(**a**,**b**) TEM and STEM images of the RG1 sample. (**c**) EDS mapping image and (**d**,**e**) elemental EDS images for Fe and O. (**f**,**g**) TEM and STEM images of the RG2 sample (RG1 applied at different adsorbent dose). (**h**) EDS mapping image and (**i**–**k**) elemental EDS images for Pb, Fe, and O. (**l**,**m**) TEM and STEM images of the RG3 sample (RG1 applied in Cumbaza river). (**n**) EDS mapping image and (**o**–**q**) elemental EDS images for Pb, Fe, and O. (**r**,**s**) TEM and STEM images of the RG4 sample (RG1 applied in Mantaro river). (**t**) EDS mapping image and (**u**–**w**) elemental EDS images for Pb, Fe, and O.

The morphological properties for the RGx series were carefully evaluated, and they are summarized in Table 3. It can be seen from Figure 4a–w that after Pb(II) removal, the γ-Fe_2_O_3_ NPs conserved their morphologies, and the mean particle sizes were similar to those expected by using a co-precipitation route, as seen by the particle size distribution shown in Figure S4. Additionally, the polydispersity values ranging from 0.3 to 0.4 for the four samples were in agreement with other samples synthesized via co-precipitation [29], indicating that the Pb adsorption naturally occurred.

**Figure 5:**
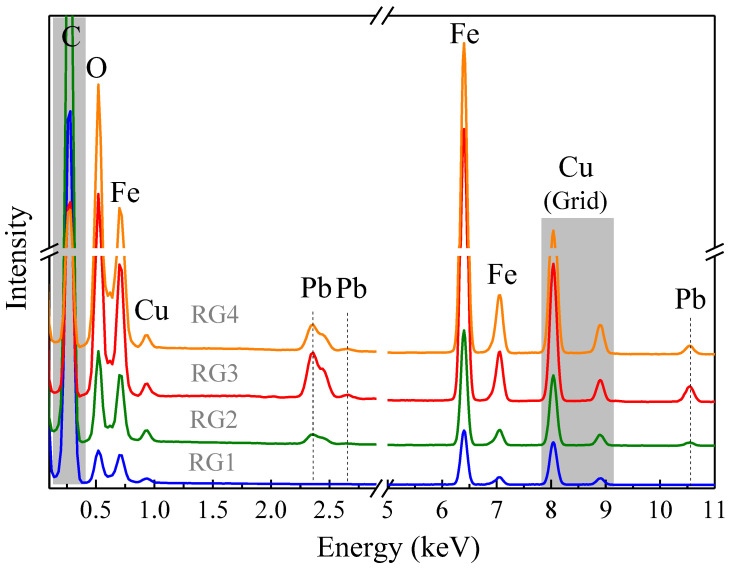
EDS spectra for the RGx series (x = 1, 2, 3 and 4). RG2 sample is the RG1 applied at different adsorbent dose, RG3 sample is the RG1 applied in Cumbaza river, and the RG4 sample is the RG1 applied in Mantaro river.

From Figure 5, the atomic percentage composition was estimated for the RGx series (x = 1–4) to lie between 0.6 and 1.5 at. %, indicating that the original RG1 sample adsorbed the Pb. As can be seen from the results in Table 4, no elements other than Fe and O were found by the EDS mapping analysis, except Pb in samples obtained after interactions of the RG1 sample with contaminated effluents. 

#### 3.8.2. Magnetic Measurements

##### VSM Analysis

**Figure 6:**
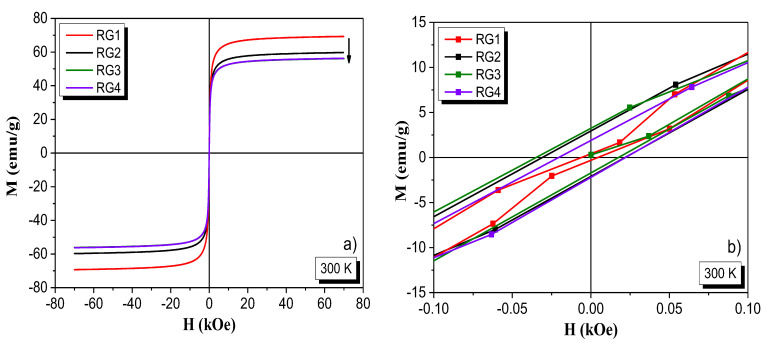
(**a**) RT *M(H)* curves for the RGx series (x = 1–4) and (**b**) zoomed region for scan field of ±0.10 kOe. In a) RG2 and RG4 are overlapping. RG2 sample is the RG1 applied at different adsorbent dose, RG3 sample is the RG1 applied in Cumbaza river, and the RG4 sample is the RG1 applied in Mantaro river.

The *M(H)* curves were measured at RT and 5 K for the RGx series, and their results are shown in Figure 6a. In addition, the FC and ZFC protocols (*M(H)* curves shown in Figure S5) were also performed to check whether the samples could show, for example, the exchange bias effect or any remarkable change. The results indicated the absence of a bias effect. On the other hand, the RG1 sample exhibited, at RT, the highest saturation magnetization (*M_s_*) at 69.2 emu g^−1^, a value that agrees with that reported for the bulk γ-Fe_2_O_3_ phase [30]. However, the samples with adsorbed Pb had a reduced *M_s_* value of 59.7 emu g^−1^ for the RG2 sample and 56.2 emu g^−1^ for the RG3 and RG4 samples (samples used in the kinetic experiments). Hence, it is clear that when Pb surface adsorption occurs, a measurable reduction in the *M_s_* values is detected, indicating a reduction in magnetic mass of the original sample (RG1). As expected, a similar trend is also observed for *M_s_* values measured at 5 K, with values for RG1 > RG2 > RG3-RG4, as estimated from Figure S4a–d. By looking into the zoomed *M(H)* curves of Figure 6b, the coercive field (*H_c_*) values are between ± 50 Oe. These small *H_c_* fields indicate weak magnetic interactions among the NPs in the RGx series, while, at 5 K, the samples clearly exhibit a ferrimagnetic-like loop (see insets of Figure S5a–d) that is associated with the γ-Fe_2_O_3_ phase. The important parameter for magnetic remediation is, however, the *M_s_* value at RT [12]. For the after-adsorption samples, we observed a reduction of ~19% of the *M_s_* value at RT. Despite this reduced *M_s_* value, it is still sufficient for remarkable Pb adsorption [12].

##### ^57^Fe Mössbauer Spectroscopy Analysis

**Figure 7:**
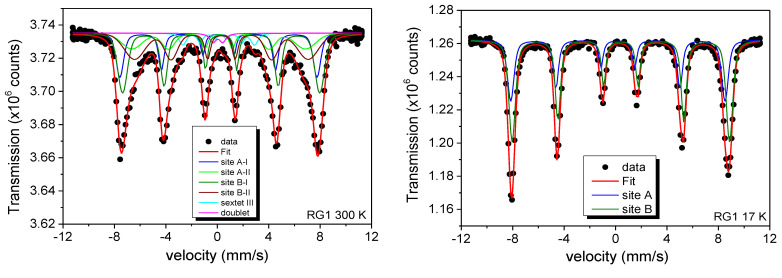

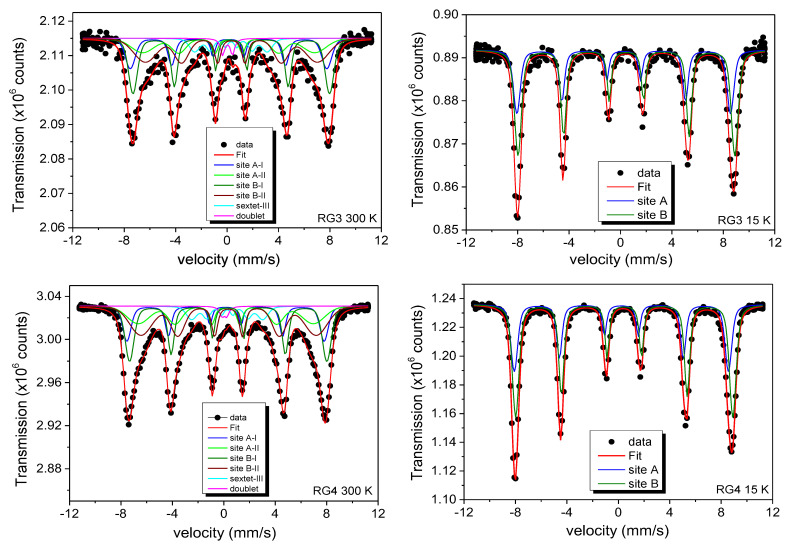
^57^Fe Mössbauer spectra for the RGx series (x = 1,3, and 4) taken at 300 K, 15 K, and 17 K. The two Fe sites (A: tetragonal (blue) and B: Octahedral (green)) of the spinel-like structure of the $\gamma -{\mathrm{Fe}}_{2}O_{3}$ are clearly shown for measurements at 15 K. The other subspectra are discussed in the text. RG3 sample is the RG1 applied in Cumbaza river and the RG4 sample is the RG1 applied in Mantaro river.

^57^Fe Mössbauer spectra were investigated, in particular, for the samples before (RG1) and after (RG3 and RG4) adsorption kinetic experiments. The spectra recorded at 15 and 17 K are displayed in Figure 7, and the results of the fits are summarized in Table 5. These spectra were fitted using two magnetic hyperfine patterns, i.e., sextets due to Fe trivalent tetrahedral (A) and octahedral (B) sites commonly found in cubic spinel structures [23]. The ratio of spectral areas of absorption for the two sites (R.A.A.(B)/R.A.A.(A)) was kept fixed to 1.666, as would be expected for the bulk γ-Fe_2_O_3_ phase. The values for center shifts CS and magnetic hyperfine fields B_hf_ of A and B sites are in excellent agreement with those reported in the literature for this phase [23].

The mean value of the nuclear electric quadrupole interaction Q could be kept at zero, as expected for γ-Fe_2_O_3_. The apparent broadening of the magnetic patterns seems clearly related to a field distribution of assumed Gaussian shape; the fits gave widths σ of about 1 T for all samples. These fits indicate that, in addition, the Lorentzian line widths are broadened even at low temperatures (about 0.38–0.39 mm/s), which can be attributed to the distribution of quadrupole shifts related to structural distortions in the nanometric sample particles.

The fits to the ^57^Fe Mössbauer spectra recorded at RT are also shown in Figure 7, and their hyperfine parameters are given in Table 6. For these fits, we required six components: (i) two sets (I and II) of magnetic hyperfine correlated components, each comprising two sextets for the A and B sites (I(A,B) and II(A,B)) with R.A.A.(B)/R.A.A.(A) spectral area ratios kept fixed at 1.666, as found for the ideal spinel structure; (ii) one extra sextet III (also with features of Fe^3+^) of lower weight (about 5–10% of total); and (iii) a doublet (also with Fe^3+^ characteristics) with even smaller fraction, which is either associated with very small grains of the sample in superparamagnetic regime at RT or due to the presence of an “pure Fe impurity”, as the XPS data suggest. Again, for all magnetic components, the quadrupolar shifts for the four sextets I (A,B) and II (A,B) were kept equal at 0. The center shift (CS) values were fixed by the relation CS(B) = CS(A) + 0.21 and, finally, the hyperfine magnetic fields were kept equal B_hf_(A) = B_hf_(B). The B_hf_ values for A and B sites are smaller for two sextets of the set II than those of sextets of the set I. The values of magnetic hyperfine fields for the RG1, RG3, and RG4 samples are practically the same for both sets of sextets I and II, and their relative spectral weights are nearly the same. The magnetic hyperfine fields again revealed a broadening effect, mainly related to an assumed Gaussian-shaped distribution with width σ. Notably, σ is larger for set II of the sextets than for set I.

In brief, both sets I and II of the four sextets (A and B) are compatible with the presence of stoichiometric γ-Fe_2_O_3_, indicating that our NPs have the well-organized atomic arrangement of a cubic spinel structure. The reduced magnetic hyperfine fields for set II compared with set I suggest a fraction with faster collective magnetic relaxation at RT [31] that could be related to smaller particles. At low temperatures, these spin fluctuations observed at RT are magnetically blocked and, therefore, the spectral contributions by both sets of particles coincide and cannot be distinguished by hyperfine interactions, i.e., only one set of the spinel hyperfine patterns is observed. Therefore, the spin relaxation effect at RT may help us to understand the magnetic features of the ensemble of the particle size distribution of our samples, showing most Fe ions in the magnetically blocked regime at RT but with some spins of small particles in the relaxation process.

Further, sextet III can only be detected in the spectra taken at RT. Its hyperfine parameters are also the same for the three samples. At low temperatures, we cannot resolve its spectral contribution, indicating that the related magnetic hyperfine field is in the range of the typical trivalent iron oxide species. The strongly reduced value of magnetic hyperfine splitting at RT suggests that this component may occur at the particle surface, where Pb adsorption will take place. Unfortunately, the present data do not allow us to further specify the nature of this Fe species. In fact, we will show below, using XPS results, that a new shell formed on the γ-Fe_2_O_3_ NPs, possibly with a type of Fe oxyhydroxide (FeOOH), but that the Mössbauer parameters of known and pure FeOOH species do not agree with those of sextet III.

In summary, the Mössbauer results suggest that the two samples (after Pb adsorption) are apparently identical if one considers the situations before and after Pb adsorption.

Notably, the Mössbauer spectra taken at 15 and RT before Pb adsorption only show trivalent magnetic Fe species as those typically found for the cubic γ-Fe_2_O_3_ phase. This appears to be in opposition with our earlier proposed core–shell model, where the Fe_3_O_4_ cores were derived from the discussion of Raman data (Section 3.4). It can, however, be understood when considering that these experiments (magnetic measurements, Mössbauer spectroscopy, adsorption experiments) were carried out, at least, two months after the structural, vibrational, and textural experiments of the as-prepared NPs, i.e., the Fe_3_O_4_ cores of the sample would be totally oxidized by this time and by the exposition to air under normal conditions [12,23]. After Pb removal, no apparent change was observed using Mössbauer spectrometry at 15 K, where a magnetically blocked state, characteristic of bigger particle sizes (>10 nm, as seen by TEM), can explain the spectra.

For improving our knowledge on the surface properties of the RGx series, XPS experiments were subsequently performed, and the results will be discussed in the next section.

#### 3.8.3. XPS Analysis (Adsorption Mechanism)

**Figure 8:**
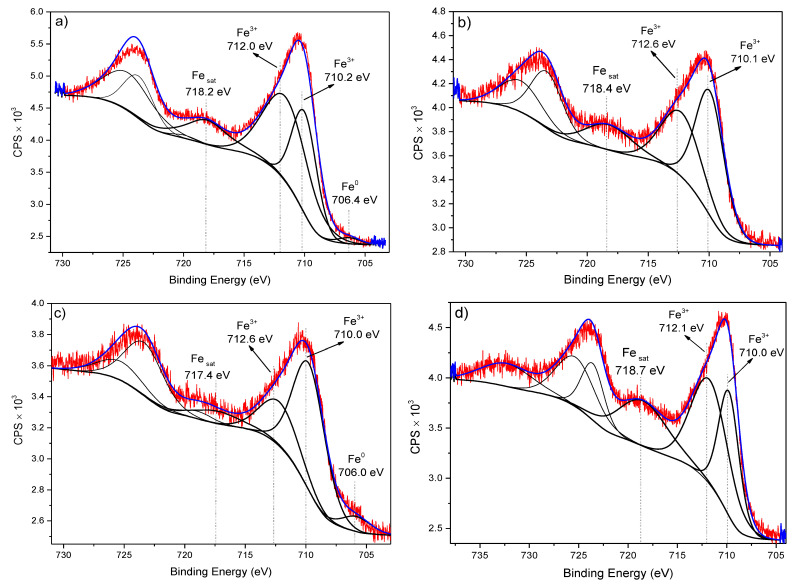
High-resolution XPS spectra of Fe2p regions for the (**a**) RG1, (**b**) RG2, (**c**) RG3, and (**d**) RG4 samples. RG3 sample is the RG1 applied in Cumbaza river and the RG4 sample is the RG1 applied in Mantaro river. Red curve is the experimental data, blue line is the fit spectra, and black lines are the fit components.

The RG1 sample showed binding energies at 710.2 eV for the Fe 2p_3/2_ level, representing an Fe^3+^ valence state, which is very close to the chemical compound of the γ-Fe_2_O_3_ phase [32]. In a γ-Fe_2_O_3_ phase, according to different authors, the peak in Fe 2p_3/2_ is positioned between 710.6 and 711.0 eV [33,34,35]. Although, in this work, the binding energy (BE) of Fe 2p_3/2_ is below 0.4–0.8 eV, the statement that this energy of 710.2 eV represents the γ-Fe_2_O_3_ is confirmed through the difference in energies between the respective peak (710.2 eV) and its satellite, which, for the γ-Fe_2_O_3_ phase, gives a value of 8.0 eV [35], as seen in Table 3 and Figure 8a. Hence, no Fe^2+^ spin state was found, suggesting a totally oxidized core in the γ-Fe_2_O_3_ phase. In the totally Pb adsorbed samples (Figure 8b–d), the BEs (principal Fe 2p_3/2_ peak) were not severely altered, indicating that the γ-Fe_2_O_3_ NPs (in their “bulk”) were not severely affected after Pb adsorption. However, it should be mentioned that there was a decrease in the atomic percentage of Fe at the NP surface, indirectly indicating that this was due to the presence of Pb-adsorbed ions.

Moreover, it should be said that there is another BE in the Fe 2p_3/2_ at 712.0 eV for the RG1 sample. It may refer to the formation of another species with Fe^3+^, and the most common chemical species should be a type of iron oxyhydroxide (FeOOH) [35,36]. It arises due to the hydrated and/or alkaline condition to which this sample was exposed [35]. Indeed, considering that the XPS is a surface technique and the amount of this chemical species is larger than that of the γ-Fe_2_O_3_ itself (see Table 7), one can infer that this hydrated species must only be found on the NP surface since ^57^Fe Mössbauer spectrometry suggested the presence of the spinel cubic structure for all RGx (x = 1, 3, and 4) series. Furthermore, the ratio of the amount of FeOOH against the total Fe_2_O_3_, being very small, prevents the detection of a sizable Mössbauer spectrum related to the FeOOH phase. We assumed that this shell is not totally homogenous at the surface due to the molecular size of the FeOOH phase. Finally, a small amount of metallic Fe (or FeO) was also identified in the RG1 sample, which could be related to the presence of the minor-intensity “impurity” doublet detected in the RT Mössbauer spectrum, as seen in Table 7. From the Pb-adsorbed samples (Figure 8b,c) [37], an increase of 0.6 eV in the Fe 2p_3/2_, representative of the FeOOH phase, was observed, which shows an alteration in its chemical state, possibly due to the incorporation of Pb in that species of hydrated Fe, especially for the RG2 and RG3 samples. Another important observation is associated with changes in the energy difference between the satellite peaks and the Fe^3+^ peak of the FeOOH species. These changes suggest that there was a greater decrease for the RG2 and RG3 samples (Figure 8b,c). This should be associated with changes in Fe^3+^ coordination due to the Pb adsorption occupying Fe^3+^ sites.

**Figure 9:**
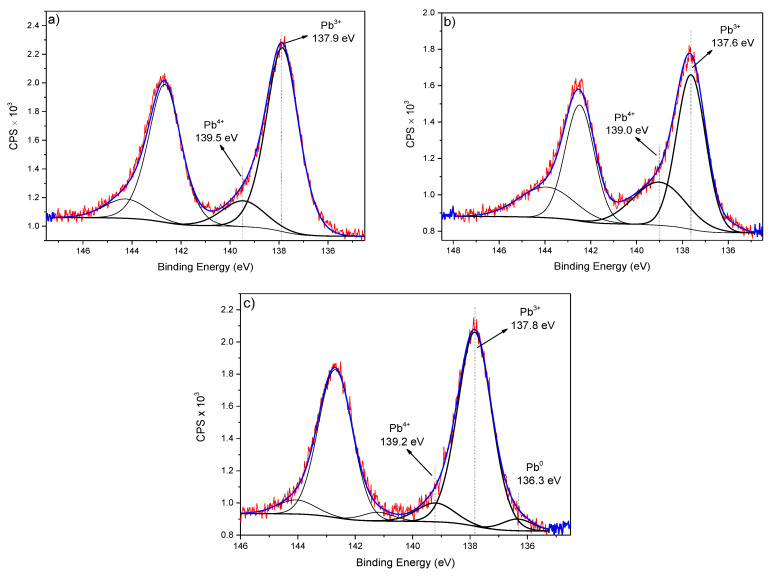
High-resolution XPS spectra of Pb4f regions for the (**a**) RG2, (**b**) RG3, and (**c**) RG4 samples. RG3 sample is the RG1 applied in Cumbaza river and the RG4 sample is the RG1 applied in Mantaro river. Red curve is the experimental data, blue line is the fit spectra, and black lines are the fit components.

On the other hand, Figure 9 shows the high-energy-resolution peaks of Pb 4f associated with the RG2, RG3, and RG4 samples. From the fit of the Pb 4f peaks of these samples, the presence of two oxidation states of Pb (Pb(II) and Pb(IV)) was determined, with a stronger contribution by Pb(II). The energy associated with Pb(II) is similar to that of a Pb oxide and, more specifically, closer to the binding energy of Pb_3_O_4_ [36,37] and can also be related to the Pb(NO_3_)_2_ complex in the modified solutions. Actually, the Pb_3_O_4_ compound is a mixed valence compound of Pb(II) and Pb(IV). This latter result suggests that Pb(IV) ions are also present in real waters, and both Pb(II) and Pb(IV) adsorption is favored by the FeOOH/Fe_2_O_3_ interface and also due to the surface deprotonation reaction of γ-Fe_2_O_3_ given by Equation (2). In addition, it is possible to observe that the ratio of the elemental amount of the FeOOH/Fe_2_O_3_ decreases from 0.72 to 0.43, respectively, for the RG2 and RG3 samples (see Table 7); likewise, a proportional atomic quantity also decreases for Pb(II) from 1.5 to 0.9 at.% for these two samples. This confirms that the Pb adsorption also occurs on the γ-Fe_2_O_3_ surface, in a close agreement with kinetic adsorption experiments, at the same time that this adsorption increases the binding energy of hydrated Fe^3+^ (see Figure 9b,c). The total Pb adsorption may have produced changes in the oxygen coordination that opened their bonds to Fe^3+^ to accept Pb(II) (see Figure S6). In the RG4 sample, an increase in the atomic ratio of the FeOOH/Fe_2_O_3_ was observed, as well as an increase in the amount of Pb(II) adsorbed (1.2 at.%), indicating that this sample also adsorbed Pb, but was unable to alter the FeOOH shell, which led us to deduce that the Pb_3_O_4_ phase was adsorbed on the shell surface.

**Scheme 1:**
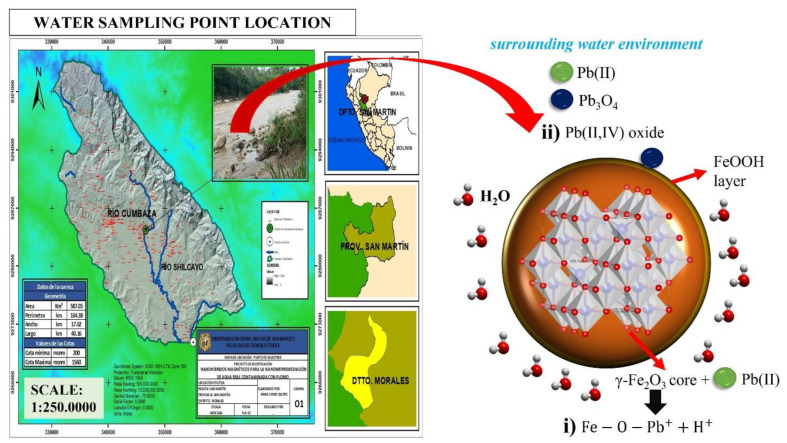
Surface adsorption mechanism for Pb removal in Cumbaza river using γ-Fe_2_O_3_ NPs.

It is worth mentioning that only XPS allowed for a direct determination of the Pb oxidation state, which is important for understanding the surface adsorption mechanism. We must also recall that the experiments were conducted in real waters where many bodies were present during the adsorption process, making it more difficult and challenging. Hence, the adsorption mechanism among the iron surface coordination phases (protecting hydrated shell) and Pb occurs favorably, as we schematically suggest in Scheme 1.

## 4. Conclusions

Initially Fe_3_O_4_ NPs were synthesized following the alkaline co-precipitation route. Structural and vibrational characterization confirmed that cubic inverse spinel structures were formed (space group Fd$\bar{3}$m). Laser power variations in Raman experiments on the RG1 sample (as-synthesized NPs) did not induce a local thermal structure transformation for intensities below 0.72 mW. However, for the after-burning protocol (>0.72 mW), the sample surface suffered transformation to the α-Fe_2_O_3_ phase. From the IR experiments with different laser power intensity, we concluded the formation of an Fe_3_O_4_-γ-Fe_2_O_3_ core–shell structure in the as-synthesized sample. This early stage revealed, however, to be chemically unstable, as proved using Mössbauer spectroscopy and XPS data, since it was oxidized by ambient air (these experiments were done months later of sample synthesis). Thus, the NPs finally employed as adsorbents were revealed to be entirely composed of the γ-Fe_2_O_3_ phase with a cubic spinel structure. The water samples for the adsorbent tests were collected from two Peruvian local rivers severely polluted with Pb. Conserved samples of the effluents from these two rivers were transported to the laboratory for batch adsorption experiments. Kinetic equilibrium times (Pb removal efficiencies) of 45 (90%) and 60 min (96%) were found for the Mantaro and Cumbaza rivers. Fe ions can also be removed using the RG1 adsorbent; the achieved values turned out to be below the permissible level of 10 µg L^−1^ in the case of Mantaro river. The adsorption rate was dominated by a pseudo-second-order kinetic model, suggesting a chemical sorption of Pb(II) ions on the RG1 surface. Hence, the adsorbent demonstrated remarkable selectivity for Pb and Fe in real waters, an effect mainly associated with their optimized textural properties (BET area of 75.9 m^2^/g and mesoporous size distribution). The optimum pH values during kinetic experiments were found to vary between 6.5 and 7.5, in agreement with the p.z.c. of the adsorbent located at 2.3 and negative zeta potential above this value. After Pb adsorption, the RG1 sample conserved its morphology, with slight variations in the PSD below 20 nm for the RG2-RG4 samples. The *Ms* values of samples after Pb adsorption had a reduction of 19% at RT but maintained a relatively high magnitude. These values indicated that the adsorbent can be reused for water remediation in subsequent cycles. Kinetic adsorption data and XPS measurements revealed two possible mechanisms for the Pb surface adsorption: (i) surface deprotonation reaction of γ-Fe_2_O_3_ NPs (above p.z.c. of 2.3) forming $\mathrm{Fe}-O-{\mathrm{Pb}}^{+}+H^{+}$ complexes and (ii) formation of a thin secondary inhomogeneous layer of FeOOH coordinating with Pb species (Pb_3_O_4_ compound). Thus, this surface chemical affinity explains the high removal efficiency in real waters, suggesting the implementation of this RG1 adsorbent as a promising nano-remediator.

## Figures and Tables

**Table 1:** Rietveld refined parameters for the RG1 sample. R_p_ (%) is the profile refinement, R_wp_ (%) the weighted profile residual, R_exp_ (%) the expected profile residual, and χ^2^ the goodness of fit.

Sample	Atoms	Atomic Positions			Thermal Parameters $\left({Å}^{2}\right)$	Lattice Parameters (Å)	Statistical Parameters
		x	y	z			
γ-Fe_2_O_3_	Fe-A	0.12500	0.12500	0.12500	0.80137	a = b = c = 8.339 α = β = γ = 90°	R_p_ = 256.0 % R_wp_ = 71.4 % R_exp_ = 68.6 % χ^2^ = 1.04
	Fe-B	0.50000	0.50000	0.50000	1.87565		
	O	0.24955	0.24930	0.24930	0.55897		

**Table 2:** Linear fit parameters obtained from the pseudo-second-order kinetic model for the Cumbaza and Mantaro rivers using the RG1 sample as adsorbent. *q_e_* is the adsorbed quantity at the equilibrium time and *k*_2_ the kinetic adsorption constant.

Sample	Cumbaza River	Mantaro River
Intercept	0.07273	1.27702
Slope	0.02931	0.34992
R^2^	0.99977	0.99947
*q_e_* (mg g^−1^)	34.2	2.7
*k*_2_ (mg g^−1^ min^−1^) × 10^−3^	12	96

**Table 3:** TEM parameters obtained for the RGx series after fitting the data with log-normal distribution. *<D>* is the mean particle size and *D_m_* the mode size obtained from the distribution. RG2 sample is the RG1 applied at different adsorbent dose, RG3 sample is the RG1 applied in Cumbaza river and the RG4 sample is the RG1 applied in Mantaro river.

Samples	<*D*> (nm)	*D_m_* (nm)	Standard Deviation	Asymmetry
RG1	12.65	11.74	3.5	0.85
RG2	17.07	15.21	5.98	1.09
RG3	16.31	14.73	5.36	1.02
RG4	14.29	12.37	5.63	1.24

**Table 4:** EDS atomic composition for the RGx series. RG2 sample is the RG1 applied at different adsorbent dose, RG3 sample is the RG1 applied in Cumbaza river and the RG4 sample is the RG1 applied in Mantaro river.

Sample	Fe (% at)	O (% at)	Pb (% at)
RG1	31.2	68.8	-
RG2	30.2	68.8	0.9
RG3	31.6	67.0	1.4
RG4	29.0	70.4	0.6

**Table 5:** Hyperfine magnetic parameters obtained from the fits of the ^57^Fe Mössbauer spectra recorded at 17 and 15 K for the RGx series. RG3 sample is the RG1 applied in Cumbaza river and the RG4 sample is the RG1 applied in Mantaro river.

	R.A.A. (%)	CS(vs Fe) (mm/s)	B (T)	σ(T)	Q (mm/s)	W (mm/s)
RG1 17K						
A	38 (2)	0.303 (5)	51.8 (2)	1.1 (1)	0.00 (2)	0.39 (1)
B	62 (2)	0.514 (5)	52.6 (2)	1.0 (1)	0.00 (2)	0.39 (1)
RG3 15K						
A	38 (2)	0.359 (5)	51.6 (2)	1.1 (1)	0.00 (2)	0.37 (1)
B	62 (2)	0.469 (5)	52.4 (2)	1.1 (1)	0.00 (2)	0.37 (1)
RG4 15K						
A	38 (2)	0.344 (5)	51.8 (2)	1.1 (1)	0.00 (2)	0.37 (1)
B	62 (2)	0.569 (5)	52.5 (2)	1.0 (1)	0.00 (2)	0.37 (1)

**Table 6:** Hyperfine magnetic parameters obtained from the fits of the ^57^Fe Mössbauer spectra recorded at RT for the RGx series. RG3 sample is the RG1 applied in Cumbaza river and the RG4 sample is the RG1 applied in Mantaro river.

	R.A.A. (%)	CS(vs Fe) (mm/s)	B (T)	σ (T)	Q (mm/s)	W (mm/s)
**RG1 300 K**						
I,A	19 (2)	0.20 (1)	47.6 (1)	1.2 (1)	0.00 (1)	0.37 (1)
I,B	32 (2)	0.41 (1)	47.6 (1)	1.8 (1)	0.00 (1)	0.37 (1)
II,A	16 (2)	0.22 (1)	42.0 (1)	4.5 (2)	0.00 (1)	0.37 (1)
II,B	27 (2)	0.43 (1)	42.0 (1)	4.5 (2)	0.00 (1)	0.37 (1)
III	5 (2)	0.30 (4)	17.4 (3)	1.0 (2)	−0.17 (5)	0.37 (1)
“impurity“	2 (2)	0.15 (4)			0.8 (1)	0.4 (1)
**RG3 300 K**						
I,A	17 (2)	0.23 (1)	47.5 (1)	1.9 (1)	0.00 (1)	0.37 (1)
I,B	28 (2)	0.44 (1)	47.5 (1)	1.6 (1)	0.00 (1)	0.37 (1)
II,A	17 (2)	0.24 (1)	41.6 (1)	5.1 (2)	0.00 (1)	0.37 (1)
II,B	28 (2)	0.45 (1)	41.6 (1)	5.1 (2)	0.00 (1)	0.37 (1)
III	8 (2)	0.42 (4)	17.5 (3)	1.9 (2)	−0.01 (5)	0.37 (1)
“impurity“	2 (2)	0.15 (4)			0.8 (1)	0.4 (1)
**RG4 300 K**						
I,A	16 (2)	0.24 (1)	47.7 (1)	1.4 (1)	0.00 (2)	0.37 (1)
I,B	26 (2)	0.45 (1)	47.7 (1)	1.6 (1)	0.00 (2)	0.37 (1)
II,A	19 (2)	0.27 (1)	42.4 (1)	4.9 (2)	0.00 (2)	0.37 (1)
II,B	31 (2)	0.48 (1)	42.4 (1)	4.9 (2)	0.00 (2)	0.37 (1)
III	7 (2)	0.37 (4)	17.2 (3)	2.0 (2)	−0.01 (2)	0.37 (1)
“impurity“	1 (1)	0.15 (4)			0.4 (1)	0.4 (1)

**Table 7:** Result of fitting the XPS peaks for the RG samples.

Sample	Level	BE (eV)	at.%	Bond Type	Ref.
RG1	O 1s	529.5	50.4	O-Metal	[35,36]
	O 1s	531.2	17.9	OH-Metal	[32]
	O 1s	533.0	11.8	OH-Organic	[38]
	Fe 2p_3/2_	706.4	0.5	Fe0	[36]
	Fe 2p_3/2_	710.2	8.1	Fe^3+^ (Fe_2_O_3_)	[32]
	Fe 2p_3/2_	712.0	11.4	Fe^3+^ (FeOOH)	[35,36]
RG2	O 1s	529.4	32.6	O-Metal	[35,36]
	O 1s	531.4	27.8	OH-Metal	[32]
	O 1s	533.0	24.2	OH-Organic	[38]
	Fe 2p_3/2_	710.1	6.3	Fe^3+^ (Fe_2_O_3_)	[32]
	Fe 2p_3/2_	712.6	4.6	Fe^3+^(FeOOH)	
	Pb 4f_7/2_	137.9	3.8	Pb^2+^ (PbOx)	[36,37]
	Pb 4f_7/2_	139.5	0.7	Pb^4+^ (complex)	[37]
RG3	O 1s	529.1	22.0	O-Metal	[35,36]
	O 1s	531.7	51.6	OH-Metal	[32]
	O 1s	533.5	13.6	OH-Organic	[38]
	Fe 2p_3/2_	706.0	0.6	Fe0	[36]
	Fe 2p_3/2_	710.0	5.9	Fe^2+^ (Fe_2_O_3_)	[32]
	Fe 2p_3/2_	712.6	2.5	Fe^3+^(FeOOH)	
	Pb 4f_7/2_	137.6	2.5	Pb^2+^ (PbOx)	[36,37]
	Pb 4f_7/2_	139.0	1.2	Pb^4+^ (complex)	[37]
RG4	O 1s	529.4	36.5	O-Metal	[35,36]
	O 1s	530.8	6.2	O-Organic	[36,38]
	O 1s	531.8	12.5	OH-Metal	[32]
	O 1s	533.2	16.6	OH-Organic	[38]
	Fe 2p_3/2_	710.0	5.9	Fe^3+^ (Fe_2_O_3_)	[32]
	Fe 2p_3/2_	712.1	8.9	Fe^3+^ (FeOOH)	[36]
	Si 2p_3/2_	100.5	10.1	Si-C	[36]
	Pb 4f_7/2_	136.3	0.2	Pb0	[36]
	Pb 4f_7/2_	137.8	2.8	Pb^2+^ (PbOx)	[36,37]
	Pb 4f_7/2_	139.2	0.3	Pb^4+^ (complex)	[37]

## Data Availability

The original data related to this research can be requested at the corresponding author’s email: juan.ramos5@unmsm.edu.pe.

## Supplementary Materials

The following supporting information can be downloaded at: https://www.mdpi.com/article/10.3390/nano13101684/s1, Figure S1: Raman spectra (after) for the RG1 sample (after burning protocol). Figure S2: (a) pH vs. time of Mantaro river, (b) pH vs. time of Cumbaza river, and (c) pH vs. RG1 adsorbent mass. Figure S3: Zeta potential measurement at various pH for the RG1 adsorbent. Figure S4: Particle size distribution histograms obtained from TEM images for the RGx series (x = 1–4). Figure S5: ZFC and FC 5K *M(H)* loops for the RGx series (x = 1–4). Field cooling values are shown in the figure. The insets depict the zoomed region for each hysteresis curve. Figure S6: High-resolution XPS spectra of O1s regions for the (a) RG1, (b) RG2, (c) RG3, and (d) RG4 samples.
