# Supplementary material for: Surface Adsorption Mechanism between Lead(II,IV) and Nanomaghemite Studied on Polluted Water Samples Collected from the Peruvian Rivers Mantaro and Cumbaza
Source: Nanomaterials (Basel). 2023 May 20;13(10):1684. doi: 10.3390/nano13101684 (PMC10224039; doi:10.3390/nano13101684)
Supplement: Supplementary file 1 [file nanomaterials-13-01684-s001.zip › nanomaterials-2339330-supplementary.pdf]

nanomaterials

MDPI

Supplementary Figures

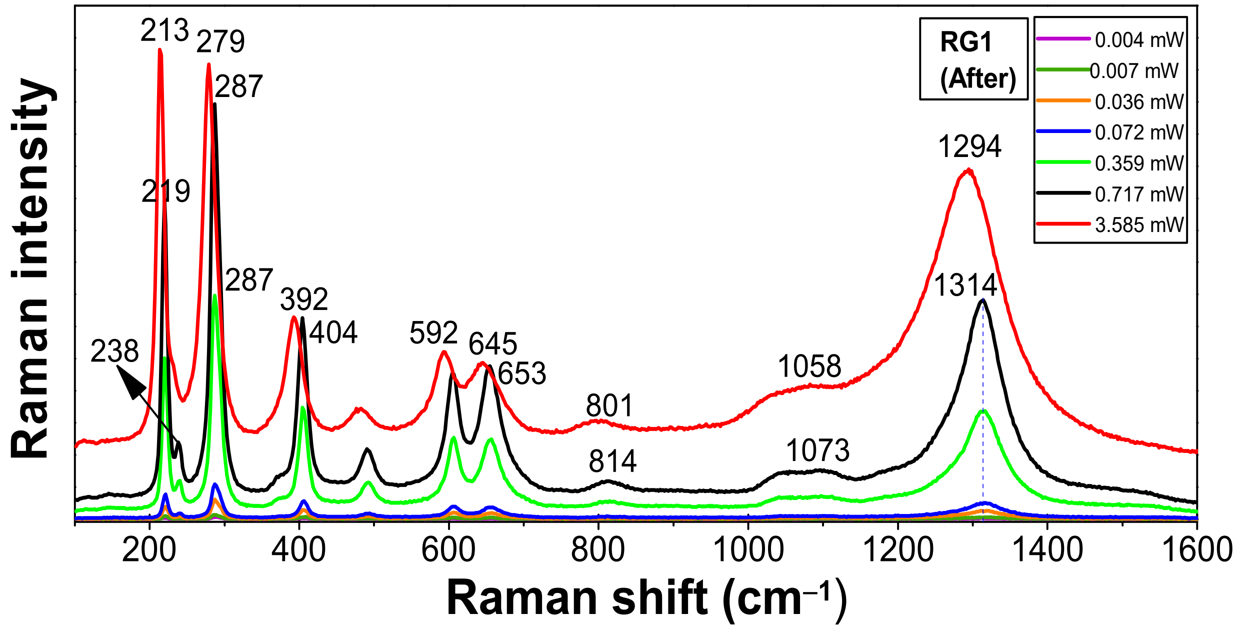

Figure S1. Raman spectra for the RG1 (after) sample (after burning protocol).

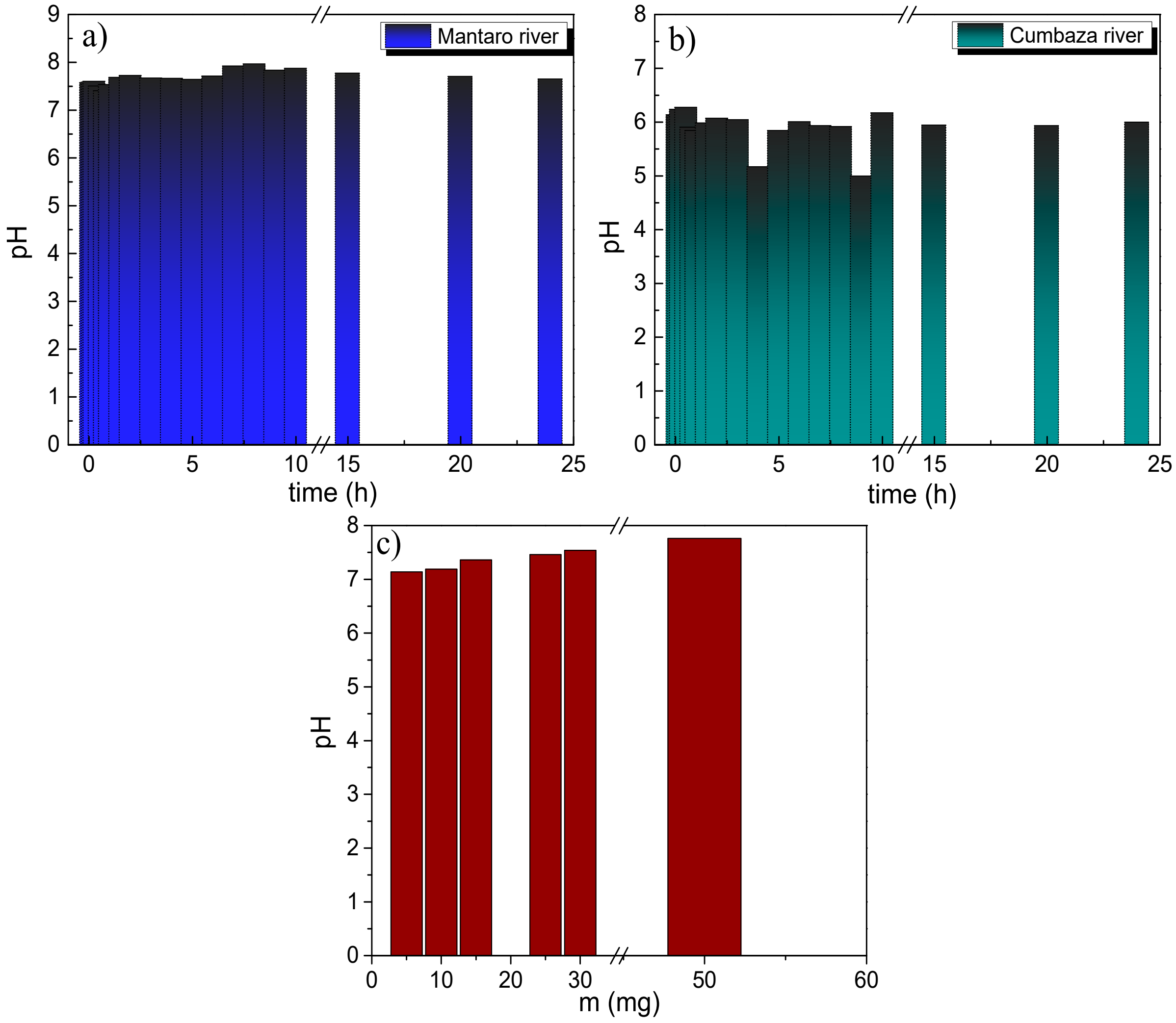

Figure S2. a) pH vs. time of Mantaro river, b) pH vs. time of Cumbaza river, and c) pH vs. RG1 adsorbent mass.

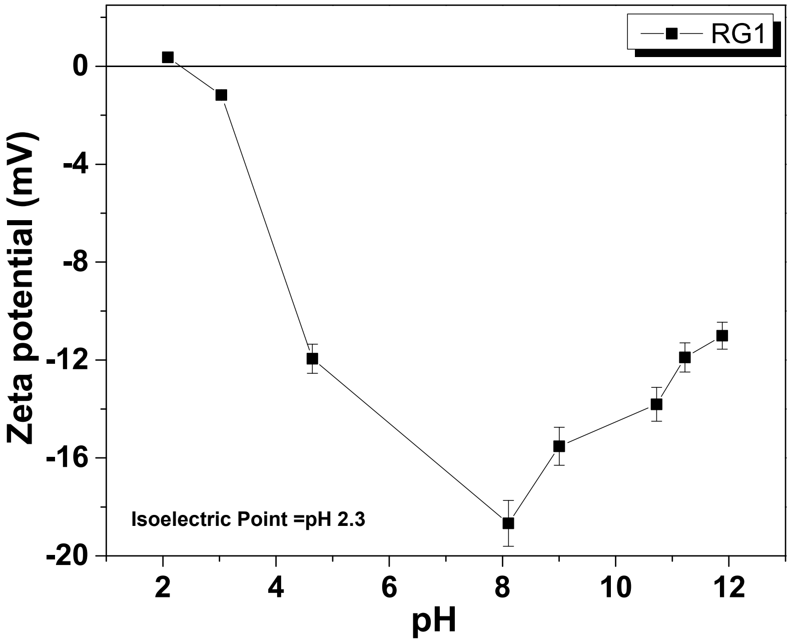

Figure S3. Zeta potential measurement at various pH for the RG1 adsorbent.

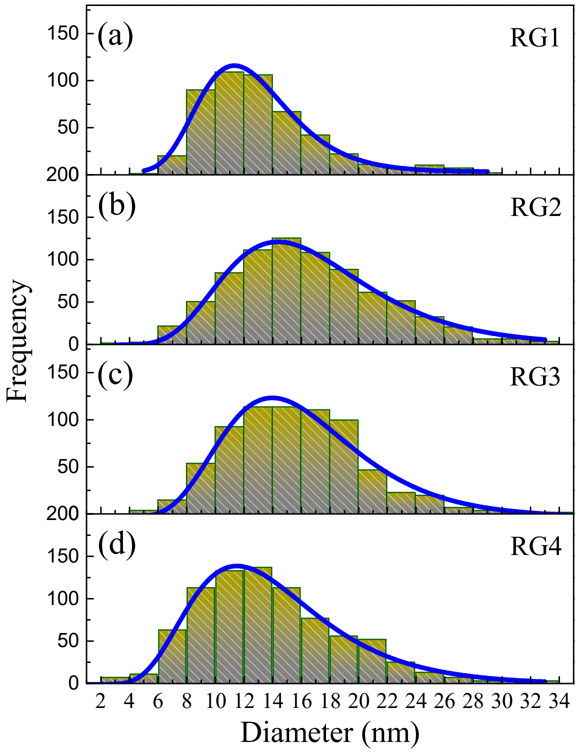

Figure S4. Particle size distribution histograms obtained from TEM images of the RGx series (x=1-4).

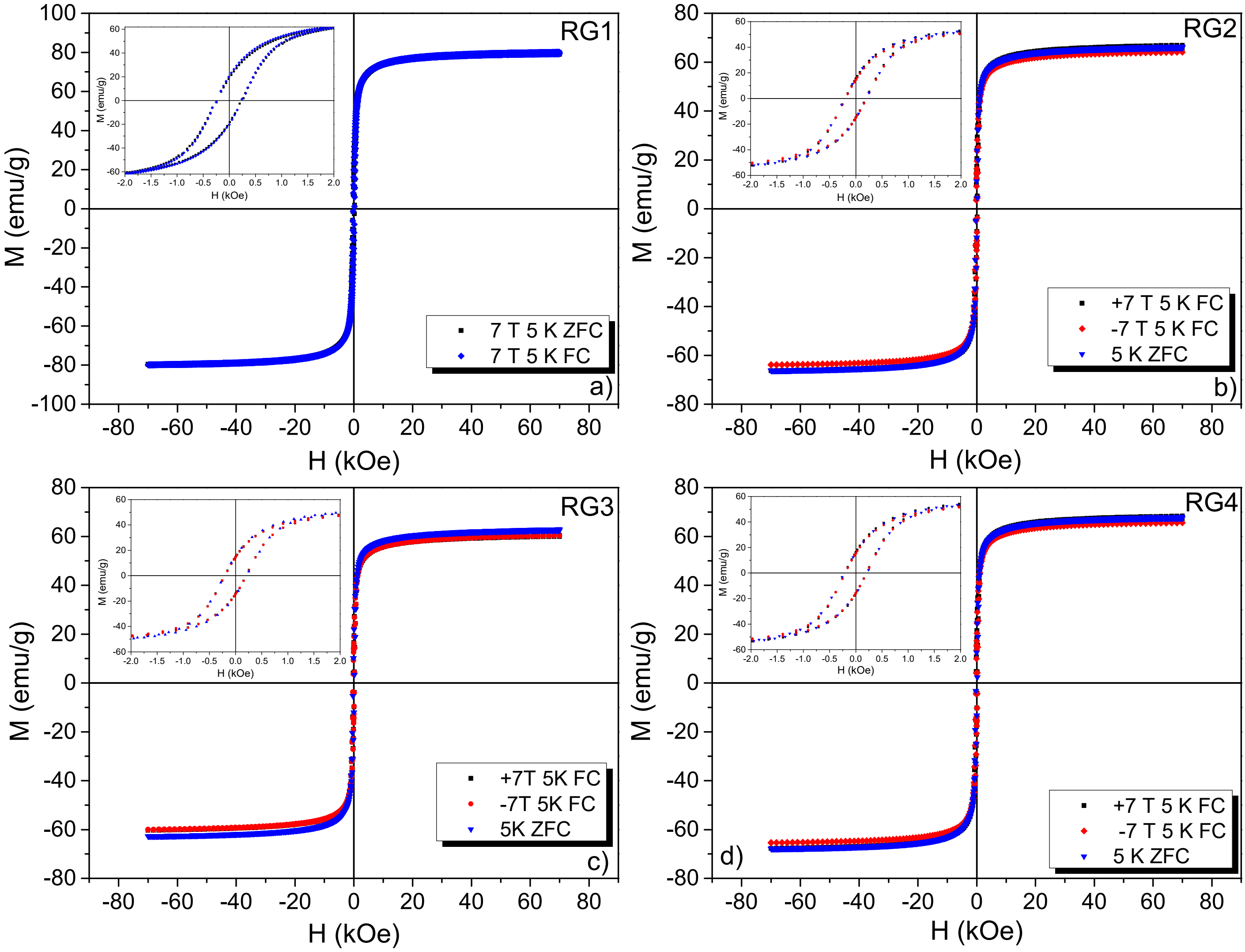

Figure S5. ZFC and FC 5K M(H) loops for the RGx series (x=1-4). Field cooling values are shown in the figure. The insets depict the zoomed region for each hysteresis curve.

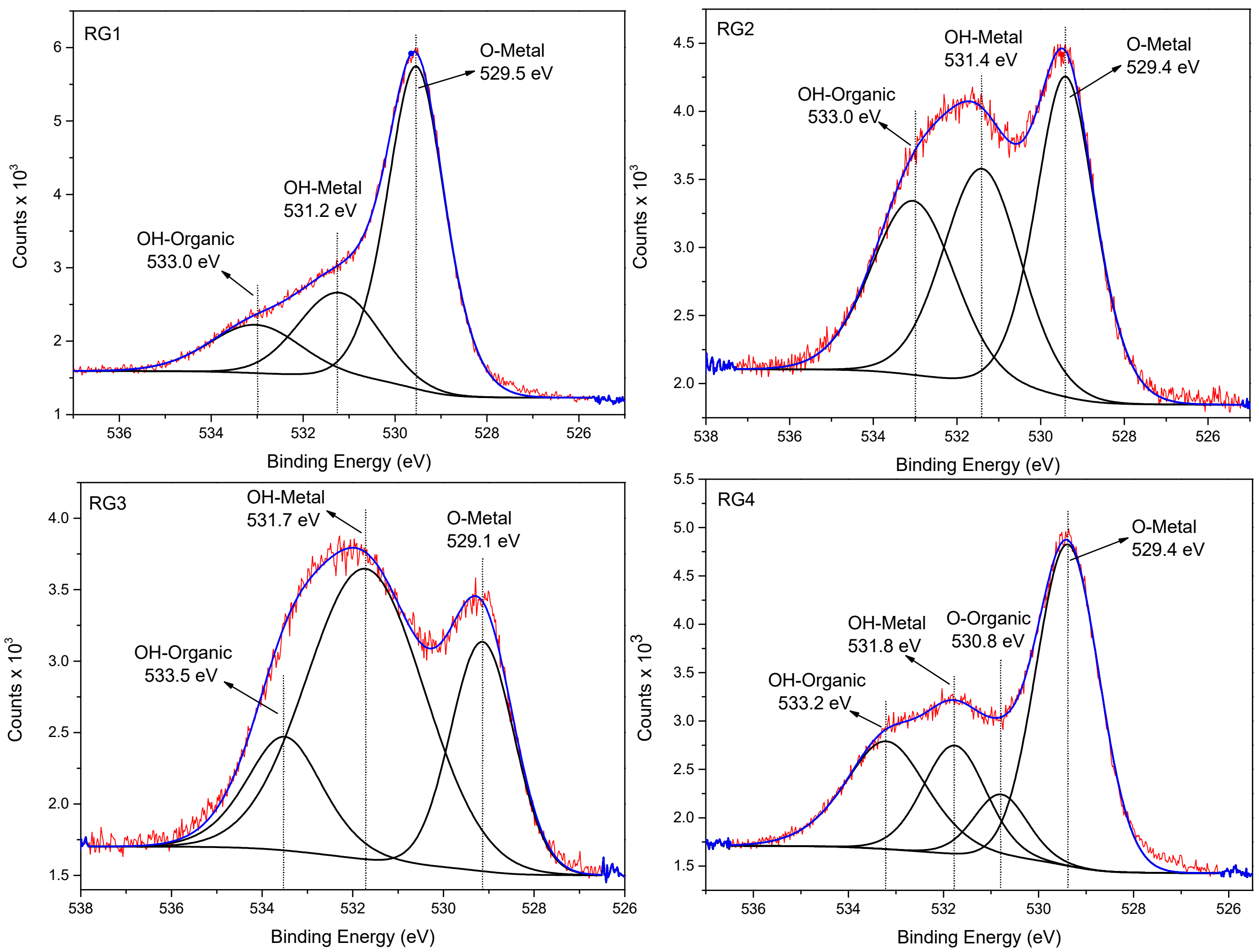

Figure S6. High-resolution XPS spectra of O1s regions for the a) RG1, b) RG2, c) RG3, and d) RG4 samples.
